# QTLs for Tolerance of Drought and Breeding for Tolerance of Abiotic and Biotic Stress: An Integrated Approach

**DOI:** 10.1371/journal.pone.0109574

**Published:** 2014-10-14

**Authors:** Shalabh Dixit, B. Emma Huang, Ma. Teresa Sta Cruz, Paul T. Maturan, Jhon Christian E. Ontoy, Arvind Kumar

**Affiliations:** 1 International Rice Research Institute (IRRI), Los Baños, Laguna, Philippines; 2 Computational Informatics, CSIRO, Dutton Park, Queensland, Australia; National Taiwan University, Taiwan

## Abstract

**Background:**

The coupling of biotic and abiotic stresses leads to high yield losses in rainfed rice (*Oryza sativa* L.) growing areas. While several studies target these stresses independently, breeding strategies to combat multiple stresses seldom exist. This study reports an integrated strategy that combines QTL mapping and phenotypic selection to develop rice lines with high grain yield (GY) under drought stress and non-stress conditions, and tolerance of rice blast.

**Methodology:**

A blast-tolerant BC_2_F_3_-derived population was developed from the cross of tropical *japonica* cultivar Moroberekan (blast- and drought-tolerant) and high-yielding *indica* variety Swarna (blast- and drought-susceptible) through phenotypic selection for blast tolerance at the BC_2_F_2_ generation. The population was studied for segregation distortion patterns and QTLs for GY under drought were identified along with study of epistatic interactions for the trait.

**Results:**

Segregation distortion, in favour of Moroberekan, was observed at 50 of the 59 loci. Majority of these marker loci co-localized with known QTLs for blast tolerance or NBS-LRR disease resistance genes. Despite the presence of segregation distortion, high variation for DTF, PH and GY was observed and several QTLs were identified under drought stress and non-stress conditions for the three traits. Epistatic interactions were also detected for GY which explained a large proportion of phenotypic variance observed in the population.

**Conclusions:**

This strategy allowed us to identify QTLs for GY along with rapid development of high-yielding purelines tolerant to blast and drought with considerably reduced efforts. Apart from this, it also allowed us to study the effects of the selection cycle for blast tolerance. The developed lines were screened at IRRI and in the target environment, and drought and blast tolerant lines with high yield were identified. With tolerance to two major stresses and high yield potential, these lines may provide yield stability in rainfed rice areas.

## Introduction

Developing crop varieties with higher yield potential is a challenging task in modern-day plant breeding. The Green Revolution led to a historic breakthrough in increasing the yield potential of crops, such as rice and wheat, through the development of semi-dwarf varieties that has fed the increasing population till now. However, it is predicted that a 60% increase in agricultural production needs to be achieved by 2050 to feed the ever-growing population [1].

Unlike other cereals, rice is cultivated across a much wider range of ecosystems, including irrigated, rainfed upland, rainfed lowland, flooded, and deepwater ecosystems across tropical and temperate climate conditions. Despite this diversity of cultivation systems and environments, the major focus of research in increasing rice yield potential has been for the irrigated ecosystem in the post-Green Revolution period. However, no major breakthrough in breaking the yield potential barrier has been achieved until now.

The low-yielding rainfed lowland rice ecosystem has great potential for yield improvement. This ecosystem occupies about 38% of the total rice area, but contributes only 21% to total rice production [2]. In Asia alone, 34 million ha of rainfed lowland rice and 8 million ha of rainfed upland rice [3] experience drought stress of varying intensity almost every year. These drought events are often coupled with increased incidence of other biotic stresses, such as blast, brown spot, and bacterial blight, leading to a further decline in rice yield. Rice yields in these ecosystems remain low at 1.0–2.5 t ha^−1^ [4].

Despite the importance of drought as a major factor in yield reduction in rainfed ecosystems, few efforts have been made to develop high-yielding drought-tolerant cultivars. As a result, a large proportion of these rainfed areas are covered with high-yielding varieties such as Swarna, Samba Mahsuri, IR64, and MTU1010 that were specifically developed for irrigated ecosystems and suffer heavy yield losses under drought. On the other hand, several drought-tolerant cultivars, such as N22 and Moroberekan, have evolved in these rainfed ecosystems through natural or artificial selection over the years. These cultivars carry genes for tolerance of a wide range of biotic and abiotic stresses, but lack farmers' and consumers' preference because of their low yield potential and poor grain quality. In many cases, a single cultivar shows tolerance of a combination of biotic and abiotic stresses. This provides a unique opportunity for rice breeders to develop high-yielding varieties with tolerance of multiple abiotic and biotic stresses.

The lack of effective selection criteria and low heritability of grain yield (GY) under drought have been reported as the two major reasons for the slow progress in breeding drought-tolerant varieties [5]. With increased precision in large-scale phenotyping, many studies have shown moderate to high heritability for GY under drought [6]–[8]. These studies used direct selection for GY in progenies derived from high-yielding drought-susceptible varieties and drought-tolerant donors as a successful strategy to improve yield under drought. This strategy has also proven beneficial to combining high yield potential with drought tolerance and has resulted in the development and release of several high-yielding rice varieties with good yield under drought in countries such as India, Nepal, Bangladesh, Indonesia, and the Philippines [9]. Along with the release of varieties through conventional breeding approaches, several large-effect QTLs for GY under drought have also been identified [2], [10]–[15]. Similarly, several studies have also reported independent and epistatic QTLs for GY and other traits of agronomic importance [16]–[19]. However, only a few studies have reported QTLs leading to a yield advantage under both drought stress and non-stress environments, taking grain yield as the primary selection criterion. Occurrence of drought is normally coupled with increased incidence of diseases such as rice blast, brown spot, and bacterial blight. However, very few studies have been undertaken to understand the genetics of these abiotic and biotic stresses simultaneously in a mapping population.

In our study, an advanced backcross (AB) QTL approach was used on a large backcross population developed from a cross between tropical japonica, drought- and rice blast-tolerant donor Moroberakan with highly popular recipient *indica* rice variety Swarna. The objectives were to: (1) develop a strategy to combine breeding approaches for tolerance of blast and drought with QTL mapping, (2) understand the effect of a selection cycle for blast tolerance during population development on segregation distortion and population structure, (3) identify QTLs for GY under drought stress and non-stress conditions, and (4) develop high-yielding blast- and drought-tolerant breeding lines from the mapping population and evaluate them in the target environment.

## Materials and Methods

Experiments for this study were conducted at the Experiment Station of the International Rice Research Institute (IRRI), Los Baños, Laguna, Philippines, during the dry seasons (DS) of 2011, 2012, and 2013 and the wet season (WS) of 2012. IRRI is located at 14°13′ N and 121°15′ E, at an elevation of 21 m above mean sea level. The study also presents results from two experiments conducted at the IRRI South Asia breeding hub (IRRI-SA) located at the International Crop Research Institute for Semi-Arid Tropics (ICRISAT), Patancheru, Hyderabad (AP), India in WS2013. ICRISAT is located at 17°31′ N and 78°15′ E at an elevation of 516 m above sea level.

### Plant material

A BC_2_F_3_-derived population developed from the cross of Moroberekan and Swarna was used in this study. Moroberekan, the tolerant donor, is an upland adapted tropical japonica [20] cultivar from New Guinea. It is a long-duration line with sturdy plant type, deep roots, and tolerance of drought and rice blast. However, this variety has poor yield potential due to its low tillering ability and lower number of grains per panicle. On the other hand, Swarna (MTU7029), the drought-susceptible recipient parent used in this study, is a lowland-adapted high-yielding indica [2], [21] variety derived from the cross Vashishtha × Mahsuri. It is a long-duration semi-dwarf variety with high tillering ability and grain yield but is highly susceptible to diseases such as rice blast and bacterial blight. This variety is grown on a large area in rainfed and irrigated ecologies across India, Nepal, and Bangladesh and is regarded as a mega-variety of rice.

**Figure 1:**
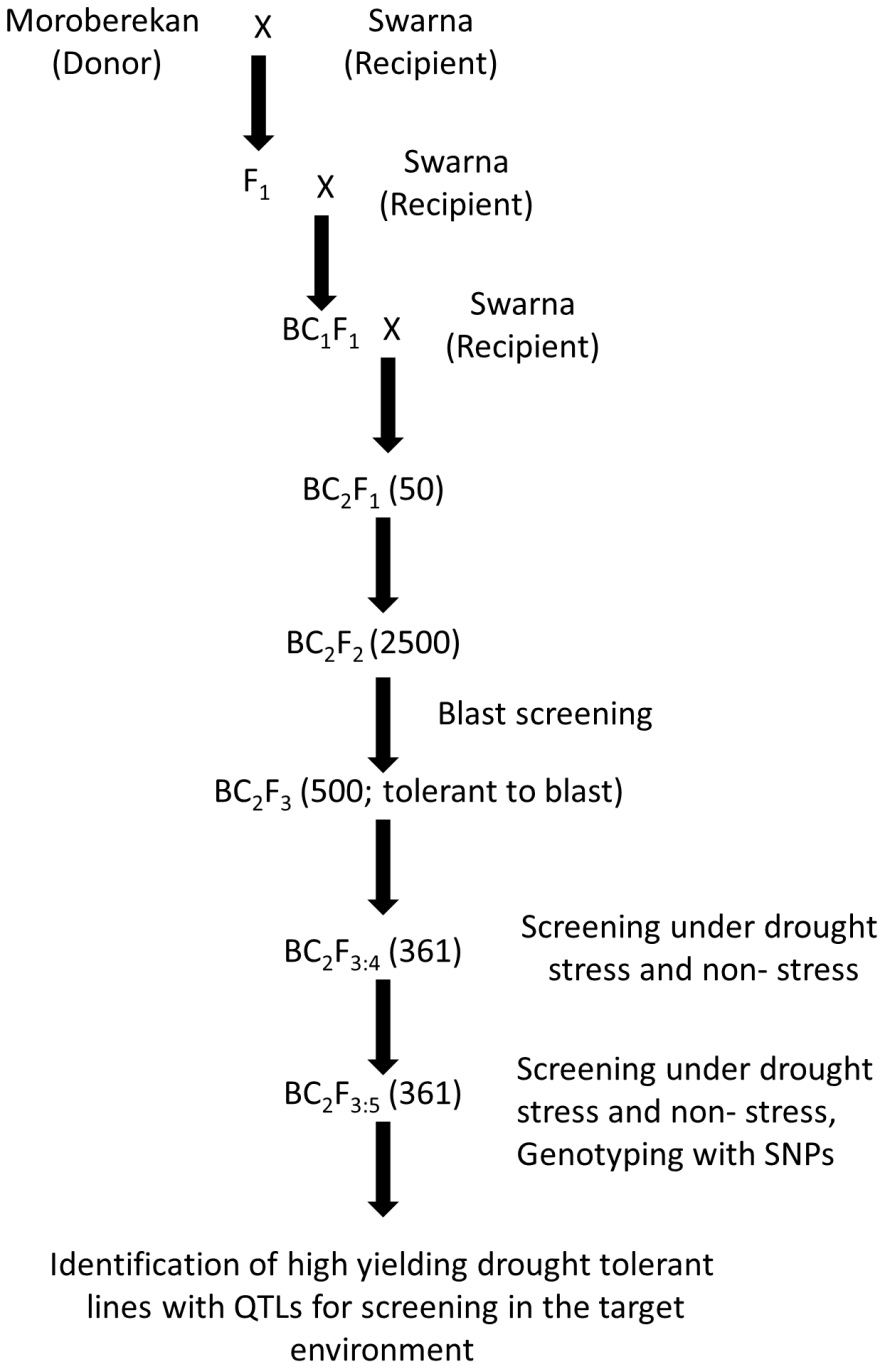
Population development and phenotypic screening strategy used to develop a blast-tolerant BC_2_F_3_-derived mapping population for mapping QTLs for high grain yield under drought stress and non-stress conditions.

Figure 1 presents the strategy used to develop the mapping population. The BC_2_F_1_ plants developed through the cross of the two parents were advanced through the single seed descent (SSD) method to develop a large BC_2_F_2_ population. This population was screened for tolerance of rice blast. Blast-tolerant plants identified through this screening were advanced to the BC_2_F_3_ generation through SSD. Seeds from BC_2_F_3_ single plants were used to develop a BC_2_F_3∶4_ mapping population to screen for GY under drought stress and non-stress conditions. Furthermore, single plant selections were made from the mapping population under drought stress. These selections were advanced, purified, and screened at IRRI, Philippines and IRRI-SA, India to identify lines that perform the best in both environments.

### Stress ecosystems

Field experiments for mapping QTLs were conducted in upland and lowland ecosystems. Throughout the study, the term “upland” refers to field experiments conducted under dry direct-seeded, unpuddled, aerobic conditions, whereas “lowland” refers to field experiments conducted under flooded, puddled, transplanted, and anaerobic conditions. Experiments were conducted under both drought stress and non-stress conditions. In this study, fully irrigated experiments with no drought stress imposed throughout the season are referred to as non-stress experiments; whereas, those experiments on which drought stress was imposed during the reproductive stage (starting at 40 and 50 days after seeding, respectively, under upland and lowland conditions) are referred to as drought stress experiments. In order to further classify the stress experiments into mild, moderate, or severe stress, the percentage reduction in yield as compared to non-stress conditions was used as a criterion [6].

### Phenotypic evaluation and data collection

Details of all the experiments, including population size, experimental design, number of replications, and plot size are presented in Table 1. A population of 361 BC_2_F_3_-derived lines was screened under lowland stress and non-stress conditions in DS2011 and DS2012. A subset of 100 lines from this population was screened under upland stress conditions in WS2012, along with parents Moroberekan and Swarna. Breeding lines developed through plant selection from this population were screened under lowland stress and non-stress conditions in DS2013 at IRRI and WS2013 at IRRI-SA. All experiments were conducted as replicated field trials in α lattice design in continuous plots. The experimental setup has proved effective for screening large rice mapping populations for grain yield under drought using smaller plot sizes. The method has been used in several QTL identification studies, which have led to the identification of large and consistent QTLs now being used in marker-assisted breeding (MAB) programs [2], [10]–[15]. Field and crop management were carried out using the protocol presented in File S1.

**Table 1:** Details of experiments conducted for identification of QTLs and screening of drought-tolerant lines identified from the mapping population at IRRI and in IRRI-SA.

Experiment	Season	Ecosystem	Population size	Location	Experimental design	Replications	Plot size
1	DS2011	LSS	361	IRRI	38×10 AL	2	1.0
2	DS2012	LMS	361	IRRI	38×10 AL	2	2.0
3	DS2011	LNS	361	IRRI	38×10 AL	2	1.0
4	DS2012	LNS	361	IRRI	38×10 AL	2	2.0
5	WS2012	USS	99	IRRI	12×10 AL	2	1.0
6	DS2013	LSS	77	IRRI	10×10 AL	2	2.0
7	DS2013	LNS	77	IRRI	10×10 AL	2	2.0
8	WS2013	LMiS	95	IRRI-SA	8×13 AL	2	2.4
9	WS2013	LNS	95	IRRI-SA	8×13 AL	2	3.2

DS  =  dry season, WS  =  wet season, LSS  =  lowland severe stress, LMS  =  lowland moderate stress, LNS  =  lowland non-stress, UMS  =  upland moderate stress.

In all experiments, data on days to flowering (DTF), plant height (PH) at maturity, and grain yield (GY) were recorded. DTF was recorded as the number of days from seeding to the day that 50% of the plants had flowering tillers. The PH of three plants from each plot were measured at maturity from the ground to the tip of the tallest tiller, and then averaged to get the mean PH for analysis. GY from each plot was harvested at physiological maturity, dried to a moisture content of 14%, and then weighed [11]. This data set was then used to calculate the quantity used in QTL analysis, namely GY of the genotypes in kg ha^−1^.

### Generation of genotypic data

Fresh leaves for all lines were collected and freeze-dried. DNA was extracted from freeze-dried leaf samples by a modified CTAB method in deep-well plates. The DNA was then quantified and purified and lines were genotyped using KASPar SNP assays. These SNPs were selected as subsets from the set of 1,536 and 44K SNP chips [22], [23] converted to SNP assays and made available through the integrated breeding platform (https://www.integratedbreeding.net/snp-marker-conversion). A total of 2,015 SNP markers were screened for polymorphism between the two parents. Out of the 2,015 SNPs screened for polymorphism, 591 polymorphic SNP loci were identified. A total of 200 polymorphic markers spaced uniformly across the genome were used to generate the genotypic data of the population. To ensure data quality, lines having percentages higher than expected of heterozygote alleles (>40%), Moroberekan alleles (>40%), or missing data (>30%) were filtered before analysis. Furthermore, markers were removed if the frequency of genotypes deriving from Moroberekan (homozygote and heterozygote) was <2%, as they were essentially monomorphic.

### Statistical analysis

#### ANOVA and means

Data from all experiments for computation of means and standard error of difference (SED) were analyzed using CROPSTAT version 7.2.3. Mixed model analysis of data from individual years was carried out using the model 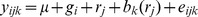
where y_ijk_ is the measurement recorded in a plot, *μ* is the overall mean, *g_i_* is the effect of the i^th^ genotype, *r_j_* is the effect of the j^th^ replicate, *b_k_(r_j_)* is the effect of the k^th^ block within the j^th^ replicate, and *e_ijk_* is the error. Genotypic effects were considered fixed and the replicates and block effects were random.

A combined analysis was conducted for lowland experiments to obtain line means across years (2011 and 2012) under stress and non-stress conditions. The model used was 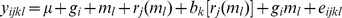
where y_ijkl_ is the measurement recorded in a plot, μ is the overall mean, *g_i_* is the effect of the i^th^ genotype, *m_l_* is the effect of the l^th^ year, *r_j_(m_l_)* is the effect of the j^th^ replicate within the l^th^ year, *b_k_[r_j_(m_l_)]* is the effect of the k^th^ block within the j^th^ replicate of the l^th^ year, *g_i_m_l_* is the effect of the interaction between the i^th^ genotype and l^th^ year, and *e_ijkl_* is the error. The effects of replicate within year and block within replicate of year were considered random, whereas the other effects were considered fixed. Due to the high variability for DTF in the population, adjusted mean GY were also calculated by taking DTF as covariate using the above models for QTL mapping. This was done to estimate the mean GY of lines free from the variation caused due to their difference in flowering time (File S3).

#### Heritability

Broad-sense heritability (*H*) of the traits for single years was calculated as shown below: 
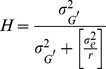
where 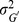 is the genetic variance, 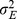 is the plot residual variance and r is the number of replications. The formula used to calculate *H* across years (2011 and 2012) was
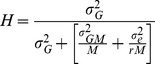

[24], where 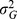 is the genetic variance, 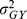 is the genotype × year variance, 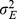 is the plot residual variance, and *r* and *M* are the number of replicates and years, respectively. Variance components were calculated using the REML algorithm of PROC VARCOMP of SAS V.9.1 [25]. A class analysis was conducted to understand the effect of DTF and PH on GY. Lines were divided into five DTF and GY classes and heat maps were constructed showing GY response in all possible combinations of these classes under drought stress and non-stress conditions. ‘Levelplot’ in R was used to construct the heat maps.

### QTL mapping

Genetic analysis of the population included estimation of segregation distortion, composite interval mapping (CIM), and estimation of epistatic effects. The level of segregation distortion was assessed using the function ‘geno.table’ in the R package qtl [26]. In order to understand the co-location of markers showing significant distortion to blast QTLs and known genes for disease resistance in rice, a physical map of SNP markers (used in this study), QTLs for blast resistance (reported in Moroberekan-derived mapping populations), and known disease resistance genes in rice was constructed. Information about disease resistance genes as well as those within the identified QTLs and their locations was obtained from the MSU Rice Genome Annotation Project, whereas QTL information was obtained from www.gramene.org and used along with the SNP markers from this study to construct a map using GGT 2 [27]. To map QTLs for GY, DTF, and PH, the genome was scanned for QTLs at a step size of 5 cM, using composite interval mapping (‘cim’) in R/qtl [26] with 5 cofactors. The genome-wide significance level was determined based on 1,000 permutations. A two-dimensional scan was also performed to identify epistatic QTLs using ‘scantwo’ in R/qtl with 1,000 permutations.

## Results

### Population development and segregation patterns

**Figure 2:**
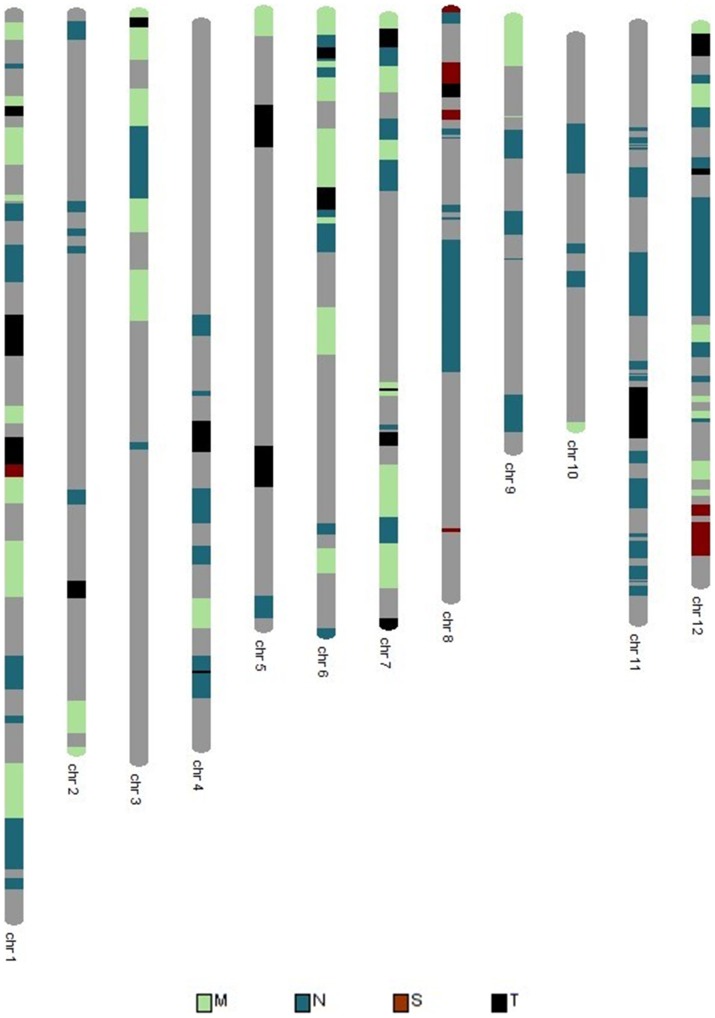
Physical map of markers showing segregation distortion, known genes for disease resistance in rice, and QTLs reported for blast resistance in the Moroberekan/CO39 population. M  =  markers showing distortion in favour of Moroberekan, S  =  markers showing distortion in favour of Swarna, N  =  genes coding for NBS-LRR proteins, and T  =  QTLs reported for blast resistance in the Moroberekan/CO39 population.

Our study reports a population development strategy using donors tolerant of more than one biotic and/or abiotic stress for simultaneous QTL mapping and pureline development in elite genetic background (Fig. 1). In this study, tropical japonica cultivar Moroberekan was used as the donor parent for tolerance of drought and rice blast, whereas *indica* variety Swarna was used as the recipient. Our strategy involved developing a large BC_2_F_2_ population and screening this population for blast tolerance. Only blast-tolerant plants were advanced further to develop the BC_2_F_3_-derived mapping population, which was used to detect QTLs for grain yield under drought and to develop purelines by plant selection in the mapping population. Since selection pressure was applied for blast tolerance, segregation distortion was expected within the mapping population. The genotypic data generated from BC_2_F_3_-derived lines were used to conduct the test for segregation distortion. A total of 50 and 9 marker loci showed segregation distortion in favour of the donor parent Moroberekan and recipient parent Swarna, respectively (Table S1). The largest segregation distortions were seen on chromosomes 3, 4, and 7 (Table S1). Segregation distortion in favour of Swarna was seen at chromosomes 1, 8, and 12. The physical map of SNP markers from this study, known disease-resistance genes, and meta QTLs for blast resistance along the rice genome, highlighted NBS-LRR disease resistance genes close to the markers showing significant segregation distortion in favour of Moroberekan on chromosomes 3, 6, and 7 (Fig. 2). Since the majority of blast resistance genes code for NBS-LRR type proteins, the presence of these genes close to the markers showing distortion may be expected as a result of phenotypic selection for blast tolerance.

### Phenotypic variation

Table 2 presents the results of ANOVA, means, heritability, and percentage yield reduction (under drought stress) in the experiments conducted at IRRI and IRRI-South Asia (IRRI-SA) under upland and lowland conditions. The full population was screened in Experiments 1-4 under stress and non-stress conditions. Yield reduction in Experiments 1 and 2 varied with stress levels in the respective seasons from severe for Experiment 1 to moderate in Experiment 2. Experiments 6 and 7 followed similar trends as Experiments 1 and 2 had in terms of yield. Both parents had similar yield under severe stress, however, Swarna (P2), being the high-yielding parent, had higher yield under moderate stress conditions in the lowland. The high yield of Swarna under moderate stress was also due to the poor adaptation of Moroberekan, an upland cultivar to lowland conditions. The population means remained higher than those for parents in both stress experiments, suggesting the role of high yield and drought tolerance in deciding the yield under drought.

**Table 2:** Means ± SED (M), broad-sense heritability (H) and P values for grain yield, days to flowering, and plant height and percentage yield reduction (YR) of trial mean grain yield under stress and non-stress conditions.

	Grain yield (kg ha^−1^)					Days to flowering					Plant height (cm)					
Experiment	P1	P2	M	H	*P*	P1	P2	M	H	*P*	P1	P2	M	H	*P*	YR
1	88	87	677±563	0.60	****	109	110	99±4	0.85	****	72	56	68±9	0.8	****	93
2	954	2326	2115±688	0.61	****	100	102	92±5	0.83	****	102	76	82±8	0.87	****	45
Combined	384	1211	1390±626	0.55	****	104	106	95±5	0.87	****	87	66	75±9	0.70	****	66
3	2234	4552	4370±868	0.78	****	97	98	91±5	0.72	****	126	91	102±8	0.84	****	
4	1248	4012	3859±1049	0.54	****	93	93	88±7	0.78	****	118	92	99±10	0.69	****	
Combined	1701	4282	4118±676	0.71	****	95	95	90±5	0.72	****	122	90	101±6	0.80	****	
5	691	89	827±357	0.92	****	95	112	100±5	0.88	****	107	75	89±7	0.85	****	80
6	0	0	1274±433	0.93	****	105	105	87±2	0.98	****	84	58	74±6	0.86	****	83
7	2893	6857	7380±1628	0.67	****	94	95	86±2	0.95	****	128	91	100±7	0.82	****	
8	NA	NA	4757±856	0.83	****	NA	NA	94±2	0.85	****	NA	NA	97±6	0.59	***	40
9	NA	NA	7895±1027	0.31	****	NA	NA	93±3	0.92	****	NA	NA	116±7	0.61	****	

a P1  =  Moroberekan, P2  =  Swarna, M  =  population mean, H  =  heritability, ****  =  significant at 0.01% *P* level.

The advantage of Moroberekan (P1) over Swarna was seen more clearly under upland conditions (Experiment 5), in which a large difference was recorded in the yield of both parents. Mild stress levels were recorded in Experiment 8 as this experiment was conducted in the wet season under naturally occurring drought stress. Moderate to high heritability estimates were recorded for yield in all experiments.

In all DS experiments, both parents had similar DTF under stress and non-stress conditions, but their progeny showed a wide range of DTF values. While Swarna is a well-known long-duration variety, there was a possibility of Moroberekan contributing to the earliness of the progeny. While, this earliness was not seen in the cultivar itself in DS experiments, this difference became clear in the WS experiment (5) when Moroberekan flowered much earlier than Swarna. Similar observations were also recorded in seed multiplication trials under lowland conditions in WS2013 (data not presented).

### Trait correlation and effect

Genetic correlations between DTF, PH, and GY under lowland stress and non-stress conditions (DS2011 and DS2012) are presented in Table 3. These four experiments were conducted using the whole population, and hence, were used to understand trait correlations. Clear correlation patterns between DTF and GY were seen in all experiments. The two traits were negatively correlated in both stress and non-stress experiments. Regardless of stress severity levels, earliness of lines related to higher yield under stress environments. Also, under non-stress conditions, a similar pattern was observed, indicating that the reduced growth period did not lead to reduced yield potential. The degree of correlation however was much higher in stress experiments than in non-stress experiments. In contrast to DTF, no clear correlation patterns between PH and GY were observed in both stress and non-stress conditions.

**Table 3:** Genetic correlation between grain yield (GY), days to flowering (DTF), and plant height (PH) under lowland drought stress and non-stress conditions.

	DTF(LSS)	PH(LSS)	GY(LSS)	DTF(LMS)	PH(LMS)	GY(LMS)	DTF (LNS I)	PH (LNS I)	GY(LNS I)	DTF (LNS II)	PH(LNS II)	GY (LNS II)
DTF(LSS)	1											
PH(LSS)	−0.597***	1										
GY(LSS)	−0.813***	0.551***	1									
DTF(LMS)	0.936***	−0.495***	−0.716***	1								
PH(LMS)	−0.042	0.727***	−0.018	−0.040	1							
GY(LMS)	−0.519***	0.168***	0.711***	−0.679***	−0.145**	1						
DTF (LNS I)	0.998***	−0.699***	−0.794***	0.869***	−0.166***	−0.568***	1					
PH (LNS I)	−0.238***	0.831***	0.185***	−0.170***	0.969***	−0.063	−0.297***	1				
GY(LNS I)	−0.297***	0.348***	0.308***	−0.318***	0.264***	0.579***	−0.403***	0.317***	1			
DTF (LNS II)	0.877***	−0.426***	−0.672***	0.845***	−0.137*	−0.664***	0.810***	−0.166**	−0.226***	1		
PH(LNS II)	0.114*	0.668***	−0.204***	0.095	0.801***	−0.238***	−0.086	0.894***	0.123*	0.037	1	
GY (LNS II)	−0.401***	0.245***	0.471***	−0.457***	0.084	0.949****	−0.482***	0.058	0.861***	−0.368***	−0.095	1

LSS  =  lowland severe stress, LMS  =  lowland moderate stress, LNS  =  lowland non-stress. *, **, ***, ****  =  significant at 5%, 1%, 0.1% 0.01% *P* levels, respectively.

**Figure 3:**
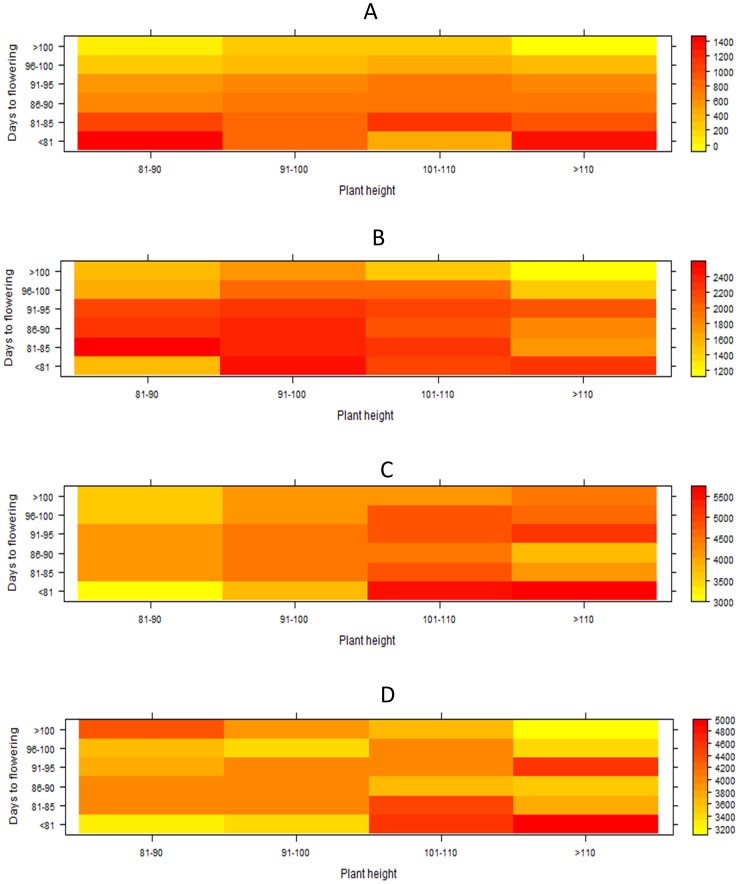
Heat maps showing the effect of DTF and PH on yield performance of lines under (A) lowland severe stress (LSS), (B) lowland moderate stress (LMS), (C) lowland non-stress (LNS I) and (D) LNS II conditions. Increasing intensity of colors from yellow to orange indicates increasing yield levels while white color shows the unavailable PH class.

In order to further understand the relationships between DTF, PH, and GY, a class analysis was conducted to measure GY for varying combinations of DTF and PH levels (Fig. 3). At any level of PH, greater differences were seen in GY for varying levels of DTF than vice versa. This agrees with the higher correlation observed between DTF and GY than between PH and GY. In general, lines with medium maturity (DTF = 81–95 days) were the most consistent at all levels of plant height across all four experiments. However, lines with plant height from 101–110 cm showed the most consistent performance across the four experiments as compared to those beyond these levels.

### Genetic variation

**Figure 4:**
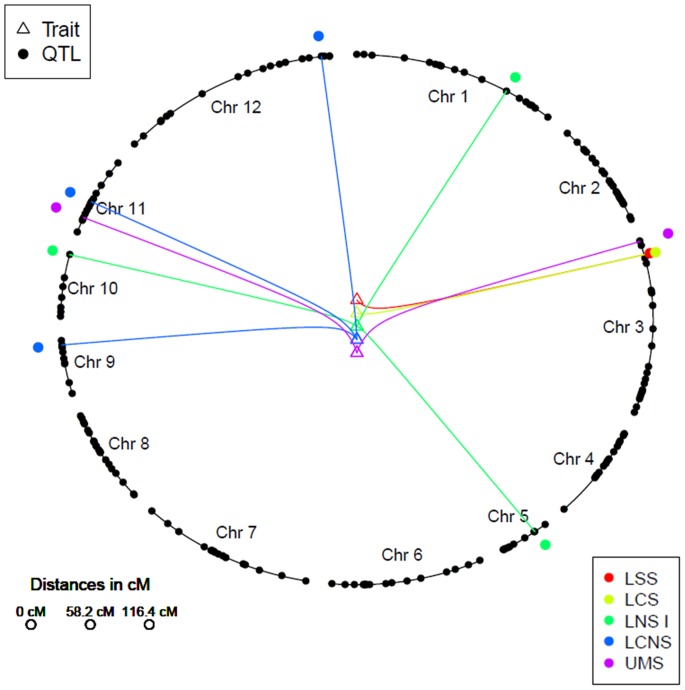
Circular genome plot showing the positions of QTLs identified for grain yield (adjusted for flowering time) under drought stress and non-stress conditions. LSS  =  lowland severe stress, LNS  =  lowland non-stress, LCS  =  lowland combined stress, LCNS  =  lowland combined non-stress, UMS  =  upland moderate stress.

Analysis of GY identified several QTLs for each trait significant within and across environments (Fig 4.,Table 4). The most consistent QTL identified for GY was *qDTY_3.2_*, which had an effect under drought stress across both upland and lowland conditions. The QTL explained a phenotypic variance effect of 16.0% and 19.0% under lowland severe stress (LSS) and upland moderate stress (UMS), respectively (Table 4). Apart from this, *qDTY_11.1_* was identified was identified under UMS condition. The QTL explained a phenotypic variance of 25.0%. To the best of our knowledge, this is the first time *qDTY_11.1_* is being reported for GY under upland drought stress.

**Table 4:** QTLs for grain yield adjusted for flowering time (GY), plant height (PH), and flowering time (DTF) identified under severe and/or moderate reproductive-stage drought stress and non-stress conditions in DS2011 and DS2012.

Trait	QTL	Ecosystem	Peak marker	Chromosome	cM	R^2^	A	*P*
GY	*qDTY_3.2_*	LSS	Id3000757	3	15.0	16.0	281.6	<0.001
	*qDTY_3.2_*	LCS	Id3000757	3	15.0	14.0	211.3	<0.001
	*qDTY_1.1_*	LNS I	id1021344	1	154.0	5.5	552.5	0.009
	*qDTY_5.1_*	LNS I	wd5002636	5	13.5	17.0	1743.0	0.009
	*qDTY_10.2_*	LNS I	id10006250	10	69.3	4.9	−350.0	0.047
	*qDTY_9.2_*	LCNS	id9007259	9	54.1	4.7	−353.4	0.012
	*qDTY_11.2_*	LCNS	id11009456	11	36.7	10.0	1223.0	0.025
	*qDTY_12.2_*	LCNS	ud12000118	12	212.0	4.8	620.7	0.050
	*qDTY_3.2_*	UMS	id3000019	3	0.0	19.0	417.6	0.014
	*qDTY_11.1_*	UMS	id11002801	11	17.4	25.0	1967.0	0.001
PH	*qDTH_1.1_*	LSS	Id1021344	1	154.0	4.2	5.1	0.003
	*qDTH_2.1_*	LMS	Id2006643	2	70.9	6.3	5.6	<0.001
	*qDTH_8.1_*	LMS	Id8007896	8	95.0	4.9	4.2	<0.001
	*qDTH_10.1_*	LMS	Id10004477	10	43.0	4.4	2.0	<0.001
	*qDTH_1.1_*	LCS	Id1021344	1	150.0	31.0	9.5	<0.001
	*qDTH_1.1_*	LNS I	Id1021344	1	154.0	9.1	9.1	<0.001
	*qDTH_2.1_*	LNS I	Id2007213	2	75.0	4.5	6.5	0.008
	*qDTH_10.1_*	LNS I	Id10004477	10	43.0	3.6	4.4	0.002
	*qDTH_2.1_*	LNS II	Id2007213	2	74.3	9.1	6.6	0.008
	*qDTH_3.1_*	LNS II	Id3002556	3	58.2	4.9	−3.3	0.031
	*qDTH_1.1_*	LCNS	Id1021344	1	154.0	9.1	7.6	<0.001
	*qDTH_2.1_*	LCNS	Id2007213	2	74.3	7.4	6.5	<0.001
	*qDTH_7.1_*	LCNS	Id7005631	7	107.0	2.8	4.6	0.024
DTF	*qDTF_3.1_*	LSS	Id3000757	3	20.0	30	−5.0	<0.001
	*qDTF_7.1_*	LSS	Id7003271	7	91.0	8.4	−5.6	<0.001
	*qDTF_10.1_*	LSS	Id10006250	10	69.3	3.4	1.9	0.040
	*qDTF_11.1_*	LSS	Id11000855	11	5.0	4.9	−2.5	0.028
	*qDTF_3.1_*	LMS	Id3000757	3	20.0	29.0	−4.9	<0.001
	*qDTF_8.1_*	LMS	Id8004692	8	60.4	13.0	−6.7	<0.001
	*qDTF_3.1_*	LCS	Id3000757	3	20.0	34.0	−5.2	<0.001
	*qDTF_8.1_*	LCS	Id8004692	8	60.4	13.0	−6.6	<0.001
	*qDTF_1.1_*	LNS I	Id1014783	1	130.0	7.9	−2.7	0.031
	*qDTF_3.1_*	LNS I	Id3000946	3	25.0	17.0	−3.4	<0.001
	*qDTF_3.1_*	LNS II	Id3000019	3	0.0	14.0	−2.6	<0.001
	*qDTF_1.1_*	LCNS	Id1014783	1	125.0	3.9	−1.5	0.023
	*qDTF_3.1_*	LCNS	Id3000946	3	25.0	21.0	−3.3	<0.001
	*qDTF_6.1_*	LCNS	Id6010576	6	118.0	7.8	−4.0	0.044
	*qDTF_2.2_*	UMS	Id2008112	2	90.0	11.0	−7.0	<0.001
	*qDTF_3.1_*	UMS	Id3000019	3	0.0	19.0	−4.2	<0.001

*qDTY*, *qDTH*, and *qDTF* are QTLs for GY, PH, and DTF, respectively. Numbers before decimals denote the chromosome number, whereas those after are the order of their detection. A  =  additive effect of the peak marker, R^2^  =  percentage of phenotypic variance explained by the QTL, LSS  =  lowland severe stress, LMS  =  lowland moderate stress, LCS  =  lowland combined stress, LNS  =  lowland non-stress, LCNS  =  lowland combined non-stress, USS  =  upland moderate stress.

In this study, large QTLs for DTF were identified, which reflect the negative correlations between DTF and GY under drought. A QTL for DTF (*qDTF_3.1_*) was also identified at the *qDTY_3.2_* locus in this study. Several other QTLs for DTF were also observed at chromosomes 2, 6, 7, 8, 10 and 11 although *qDTF_3.1_* remained to be the most consistent QTL across drought stress and non-stress conditions (Table 4). Similarly, for PH, several QTLs were identified across the genome with *qDTH_1.1_* as the largest and most consistent QTL for the trait (Table 4). Majority of the GY QTLs under stress and non-stress were contributed by Moroberekan; however, two QTLs (*qDTY_9.2_* and *qDTY_10.2_*) for yield under non-stress were also contributed by Swarna. Similarly, for PH and DTF, majority of the QTLs were contributed by the donor parent Moroberekan, but one QTL for each trait (*qDTH_3.1_* for PH and *qDTF_10.1_* for DTF) was also contributed by the recipient Swarna.

While trangressive segregants were observed in the population for all three traits, the contribution of both parents to higher performance of progeny lines and the presence of epistatic effect have become clear. In order to further explain the phenotypic variance present for grain yield under drought stress and non-stress conditions, tests for epistatic interactions were done. Epistatic interactions for GY under non-stress were observed between several loci under non-stress conditions. However, *eQTL_10.1_* consistently showed epistatic interactions with other loci across the genome (Table 5). Other consistant loci include *eQTL_1.1_* and *eQTL_8.1_* at chromosomes 1 and 8 respectively (Table 5). The high and consistent epistatic interactions of these loci suggest their importance in explaining the genetic basis of GY in lines under non-stress conditions. In this study, lines with high yield under both drought stress and non-stress conditions were identified (Table S2). Despite being more early-maturing than the recipient parent Swarna, these lines were able to maintain their yield potential equal to or more than Swarna under non-stress conditions.

**Table 5:** Epistatic QTLs affecting grain yield (kg ha^-1^) adjusted for flowering time under drought stress and non-stress conditions in DS2011 and DS2012.

Experiment	*QTL 1*	Chr (1)	cM (1)	Marker (1)	*QTL 2*	Chr 2	cM (2)	Marker (2)	*P*	R^2^
LNS I	*eQTL_8.1_*	8	75	id8006997	*eQTL_10.1_*	10	55	id10004477	0.001	11.9
LNS II	*eQTL_1.1_*	1	200	id1010697	*eQTL_10.1_*	10	45	id10004477	0.015	13.6
LNS II	*eQTL_1.1_*	1	200	id1010697	*eQTL_12.1_*	12	175	id12008700	0.037	14.7
LCNS	*eQTL_4.1_*	4	55	id4001882	*eQTL_9.1_*	9	40	id9002357	0.039	10.8
LCNS	*eQTL_7.1_*	7	80	id7002907	*eQTL_10.1_*	10	50	id10004477	0.021	8.8
LCNS	*eQTL_8.1_*	8	75	id8006997	*eQTL_10.1_*	10	55	id10004477	<0.001	12.1
LCNS	*eQTL_10.1_*	10	50	id10004477	*eQTL_8.1_*	11	25	id11006588	0.024	8.9

LNS  =  lowland non-stress, LCNS =  lowland combined non-stress, A  =  additive effect, R^2^  =  phenotypic variance explained.

**Figure 5:**
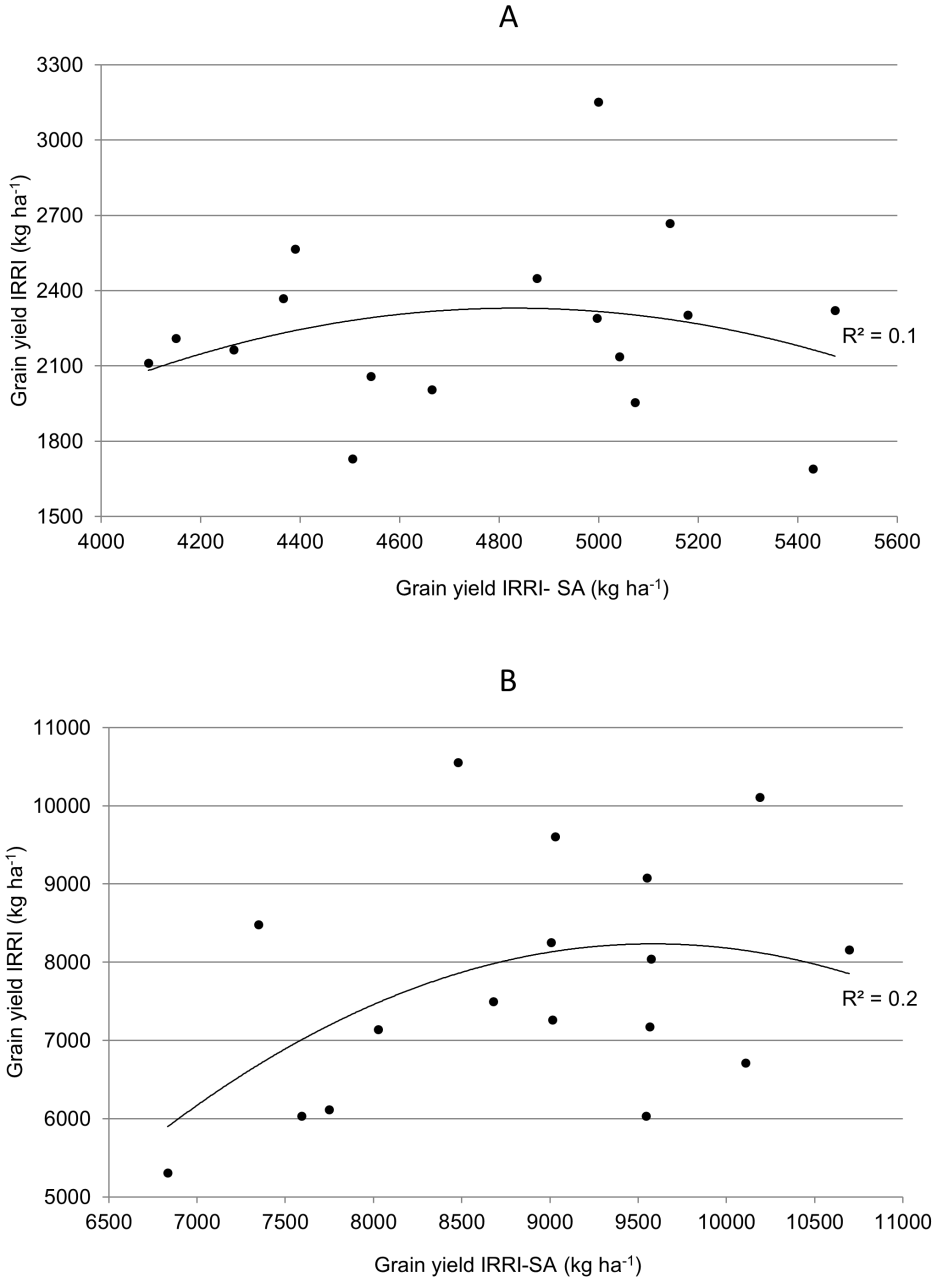
Correlation of GY performance of selected lines under drought stress and non-stress conditions in IRRI and IRRI-SA.

Lines identified from the mapping population were evaluated in the target environment (IRRI-SA) for GY under naturally occurring drought stress and non-stress conditions to understand the consistency of performance of the lines. Figure 5 presents the scatter plots between the GY of selected lines at IRRI and IRRI-SA. Although the stress levels were very mild, the high-yielding drought-tolerant lines selected in DS2013 at IRRI were able to hold similar yield patterns at IRRI-SA. The R^2^ values were non-significant, but no negative correlations were observed (Fig. 5). More significant correlations may have been apparent under higher severity of stress in the target environment. Lines finally identified from these experiments will undergo another testing for tolerance to blast to check for any loss of tolerance and grain quality testing to shortlist a final set of lines for deployment in the target environment.

### Gene content of *qDTY_3.2_* and *qDTY_11.1_*

**Figure 6:**
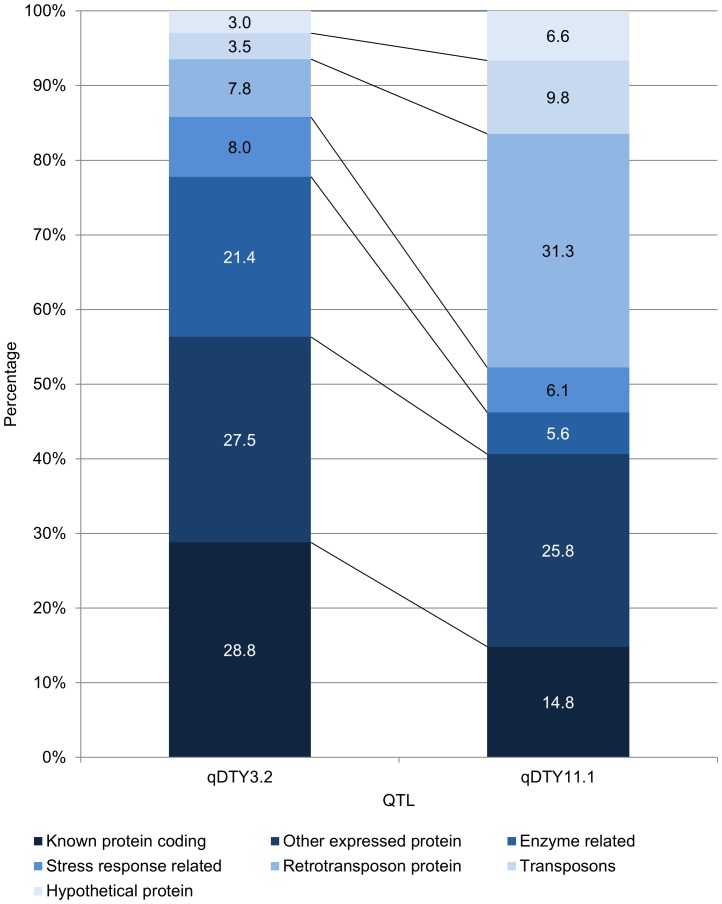
Comparison of gene content of *qDTY_3.2_* and *qDTY_11.1_* based on percentage of genes belonging to seven gene classes within the two QTLs.

Gene content of *qDTY_3.2_* **and** *qDTY_11.1_* was classified into 7 classes and percentage of genes falling into each class was calculated (Fig 6.). A large proportion of the genes in bot QTLs were genes coding for known proteins other than those known to be directly related to stress response. Apart from this, large proportion of genes were also related to other expressed proteins in both QTLs. *qDTY_3.2_* however showed a much higher percentage of genes related to enzyme synthesis and function while *qDTY_11.1_* showed a much higher percentage of retrotransposon proteins as compared to *qDTY_3.2_*. 8% of the genes within *qDTY_3.2_* 6% of genes for *qDTY_11.1_* were genes known to be directly associated to stress response or those which could be crucial for maintenance of plant function under stress. These included genes coding for heat shock proteins, no apical meristem proteins, membrane, cell cycle related proteins and zinc finger proteins (File S2). qDTY*_3.2_* also showed the presence of flowering related genes such as AP2 domain containing proteins and MADS box family genes while *qDTY_11.1_* showed the presence of a large number of nucleotide- binding site leucine- rich repeats (NBS-LRR) genes which are known to be major candidates for blast resistance.

## Discussion

Our study reports an integrated strategy to develop drought- and blast-tolerant lines along with simultaneous identification of QTLs for GY under drought. The study also attempts to explain the genetics behind the selection cycle for blast tolerance and its effect on drought tolerance. Several previous studies have used different kinds of mapping populations and strategies to identify QTLs for tolerance of rice blast and grain yield under drought. However, to the best of our knowledge, there has been no attempt to identify QTLs for high grain yield under drought stress on a mapping population tolerant of rice blast while simultaneously developing high-yielding, drought-tolerant breeding lines. Ballini et al. [28] conducted a meta-analysis to pinpoint the location of major genes reported for blast tolerance in rice. Similarly, Swamy et al. [29] conducted a meta-analysis on QTLs for grain yield under drought and reported several meta-QTLs controlling the trait across the rice genome. Both studies provide an extensive coverage of literature, indicating the large number of studies targeting these traits independently. However, reports of the two traits targeted in the same study seldom exist. In our study, the test for segregation distortion in the population showed most of the distortion to be toward the blast-tolerant parent Moroberekan, showing that it was the major contributor for blast tolerance in the population. Interestingly, distortion in favour of Swarna was also observed at some loci. While some of these marker loci co-locate with NBS-LRR genes, it is likely that these regions play a role in imparting tolerance to blast. The breeding strategy not only allowed us to select for but also to detect loci with small and large effects contributed by both parents. With observations of increased incidence of diseases such as blast in drought conditions, it becomes important to develop lines tolerant to both traits to have more stable yields in rainfed environments. Identifying QTLs independently affecting two different traits and pyramiding them together is a two-step process that is normally used to accomplish this task. The alternative mapping strategy presented in Figure 1 allows the simultaneous achievement of both these goals, particularly for traits where QTLs/genes conferring tolerance are unknown. We observed that, through systematic early-generation screening of large populations, lines with higher tolerance of one of the two targeted traits (blast in this study) may be obtained. These lines can then be used as mapping populations for QTL identification for the second target trait (high GY under drought stress and non-stress in this study), thus enabling the identification of QTLs and development of high-yielding lines with tolerance of multiple stresses in a reduced time span. Despite the segregation distortion due to screening for blast tolerance, high genetic variation could be seen for DTF, PH, and GY under drought and non-stress conditions. Clear pattern of genetic correlations between the three traits were observed and specific DTF and PH classes showed GY advantage under different stress and non-stress conditions (Table 3, Fig. 3).

Advanced backcross (AB) lines were particularly suitable in such cases where a traditional cultivar is used as a donor for an elite recipient background. Moroberekan has long been known as an important donor for tolerance of drought. In addition, being a tropical japonica accession, it is likely to carry genes that may provide opportunity to increase yield potential under normal situations. Despite these advantages, it has not been successfully used in the breeding program because of its poor combining ability and probable adverse linkage drag. In this study, the earlier segregating generations, such as F_1_- and BC_1_-derived populations, were not found suitable for the study because of the high percentage of lines with poor performance and poor plant type. However, the BC_2_-derived lines showed a very high recovery of recipient parent Swarna. In our evaluation, several lines with 10–20 days earlier duration than Swarna, but that had higher yield than Swarna under both irrigated control and drought conditions, were identified (Table S2). DS screening at IRRI has proven highly beneficial in uniform screening of large segregating populations to identify high-yielding drought-tolerant lines. Moderate to high correlation has been reported previously between yield in DS screening at IRRI and WS screening in India [30]. Similar patterns were also seen for the lines selected in this study (Figure 5).

*qDTY_3.2_* was the most consistent QTL identified for GY under drought in this study (Table 4, Figure 4). This QTL has been reported in several previous studies for showing independent or epistatic effects on GY under upland and lowland conditions [11], [13], [31], [32]. The QTL region coincides with the *HD9* locus, which controls DTF in rice. While *qDTF_3.1_* was found to collocate with this locus in the present study, it is likely that the Moroberekan allele at this locus causes earliness, leading to yield advantage under drought.

The role of drought escape mechanism coming into play due to this locus cannot be denied. However, the large variety of genes directly or indirectly related to stress response at this locus (File S2) shows that this locus could affect other traits related to stress response apart from flowering. Several existing reports also suggest the effect of this locus on a wide range of traits. Yadav et al. [13] reported the effect of this locus on grain yield under drought while Bernier et al. [10] reported its effect on plant height and biomass. Similarly, the region has been reported to affect traits including chlorophyll content, root volume under drought, low-temperature germination, and spikelet fertility [32]–[34]. The wide range of QTLs detected at this locus for traits related to yield and adaptation to abiotic stresses indicates the existence of higher genetic diversity on this chromosomal region. These results are suggestive of either multiple-linked QTLs or pleiotropic effects of one or more than one QTL on a wide range of traits.

The high genic diversity along with the effect of this region on flowering time may be the reason of its effect on yield under drought under wider range of environments as compared to *qDTY_11.1_* which showed effect under upland conditions only.

Similar to *qDTY_3.2_*, *qDTY_11.1_* also showed the presence of a wide range of stress response related genes (File S2). However, NBS-LRR genes were by far the most dominating group within this region, while these genes are well known candidates for major blast resistance genes [28]; it is likely that the QTL may affect tolerance to blast along with affecting yield under upland drought.

Epistatic interactions are one of the major factors that determine the phenotypic expression of a mapping population [35]. It has also been seen in a large number of QTL mapping studies conducted in the past that, despite nearly complete coverage of the chromosomes, the identified QTLs do not explain the entire phenotypic variance observed for a trait [35]. This potentially indicates the presence of epistatic interactions contributing to phenotypic variation in a segregating population along with other factors contributing the same. The presence of transgressive segregants in mapping populations is also suggestive of epistasis. In this study, transgressive segregants for all three traits were observed. The study of epistatic interactions showed the presence of loci having significant interactions with several other loci across the genome. These loci could be very important in understanding the genetic basis of yield under drought and non-stress conditions. Understanding the genetic composition of these loci and their effect on plant function may allow us to use these loci for maximum advantage in breeding programs.

Our study involved a two-way approach to develop lines tolerant of blast and drought with high yield potential. Some of these lines show plant type and grain type similar to Swarna with added tolerance of drought and blast. These lines may act as a replacement of the variety where Swarna is highly preferred. On the other hand, lines with different plant and grain types can be tested as prospective new high-yielding cultivars. In addition, lines with the identified QTLs are also being used in other marker-assisted breeding programs as improved donors. The study has allowed us to demonstrate the combination of conventional breeding approaches with QTL identification and to understand the effect of selection pressure on QTL mapping. This strategy can be very effective in cases where traits are likely to be controlled by multiple genes/QTLs across the genome. In such cases, phenotypic selection for one trait is less likely to affect the other, leaving sufficient variation in the population for QTL mapping.

## Supporting Information

Table S1 **Markers showing significant segregation distortion across the genome with the direction of distortion.**
(PDF)

Table S2 **Grain yield performance of lines selected under severe stress and non-stress conditions in dry-season screening at IRRI, and under naturally occurring wet-season drought and non-stress conditions at IRRI-SA.**
(PDF)

File S1 **Standard evaluation system used for large scale screening of rice lines to determine their tolerance reaction rice blast (A), field management of lowland experiments with details of crop cultivation, fertilizer management, and management of insect pests and weeds (B).**
(PDF)

File S2 **List of genes with possible function related to biotic and abiotic stress response within** ***qDTY_3.1_*** **,** ***qDTY_11.1_*** **.**
(XLSX)

File S3 **Phenotypic and genotypic data used for QTL mapping including trait values, marker names, positions and genotypes for individual lines in the mapping population.**
(XLSX)
