# Supplementary material for: QTLs for Tolerance of Drought and Breeding for Tolerance of Abiotic and Biotic Stress: An Integrated Approach
Source: PLoS One. 2014 Oct 14;9(10):e109574. doi: 10.1371/journal.pone.0109574 (PMC4196913; doi:10.1371/journal.pone.0109574)
Supplement: Table S1 — Markers showing significant segregation distortion across the genome with the direction of distortion. (PDF) [file pone.0109574.s001.pdf]

Chromosome	Marker	Position	LogP (SD)	Favoured allele
1	id1001073	7.7	19.3	M
1	id1003559	47.5	57.1	M
1	id1006772	72.3	15.1	M
1	id1004849	78.8	12.5	M
1	wd1001450	98.0	10.4	S
1	id1012784	108.9	10.1	M
1	id1014783	127.4	45.2	M
1	id1021344	154.1	21.2	M
1	id1010697	204.9	17.5	M
2	id2015558	111.7	20.7	M
2	id2015233	114.7	10.7	M
3	id3000019	0.0	124.9	M
3	id3000090	5.1	108.9	M
3	id3000757	19.5	150.0	M
3	id3000946	25.1	146.4	M
3	id3002606	55.0	50.0	M
3	id3002556	58.2	69.4	M
3	id3003294	72.2	56.5	M
3	id3005216	97.5	12.9	M
3	id3006808	116.8	36.0	M
4	id4009390	102.0	120.1	M
5	id5000045	0.0	14.0	M
6	id6000009	0.0	69.1	M
6	id6000859	12.5	71.7	M
6	id6001960	26.2	52.4	M
6	id6002884	35.9	65.3	M
6	id6004029	61.3	19.4	M
6	id6004850	75.3	69.3	M
6	id6006336	90.4	25.0	M
6	id6009055	96.1	15.2	M
6	id6014475	149.9	23.5	M
7	id7000063	0.0	3.0	M
7	id7000477	24.9	22.0	M
7	id7001246	47.3	25.1	M
7	id7002801	62.9	33.6	M
7	id7002848	64.8	38.6	M
7	id7004930	137.5	79.3	M
7	id7004343	151.5	127.1	M
7	id7003702	172.0	67.8	M
8	id8000011	17.2	16.5	S
8	id8000944	29.6	12.1	S
8	id8001604	36.3	11.0	S
8	id8006881	81.8	10.1	S
9	id9000064	0.0	89.4	M
9	id9001308	12.4	28.8	M

10	id10006250	69.3	44.9	M
12	id12002057	0.0	97.2	M
12	id12001582	13.8	44.2	M
12	id12005428	37.6	20.1	M
12	id12006198	44.7	75.4	M
12	id12006515	49.9	51.3	M
12	id12000292	87.8	33.1	M
12	id12007577	127.0	58.3	M
12	id12007161	137.9	39.8	M
12	id12007988	161.9	20.0	M
12	id12008347	170.3	14.9	S
12	id12008700	176.1	10.9	S
12	id12009243	193.8	11.8	S
12	id12009407	200.5	16.8	S
