# Supplementary material for: QTLs for Tolerance of Drought and Breeding for Tolerance of Abiotic and Biotic Stress: An Integrated Approach
Source: PLoS One. 2014 Oct 14;9(10):e109574. doi: 10.1371/journal.pone.0109574 (PMC4196913; doi:10.1371/journal.pone.0109574)
Supplement: Table S2 — Grain yield performance of lines selected under severe stress and non-stress conditions in dry-season screening at IRRI, and under naturally occurring wet-season drought and non-stress conditions at IRRI-SA. (PDF) [file pone.0109574.s002.pdf]

Line	IRRI						IRRI-SA					
	**DTF-S**	**PH-S**	**GY-S**	**DTF-NS**	**PH-NS**	**GY-NS**	**DTF-S**	**PH-S**	**GY-S**	**DTF-NS**	**PH-NS**	**GY-NS**
IR 91648-B-117-B-1-1	81.9	77.0	2566	83	86	5309	92	92	4390	89	98	6835
IR 91648-B-1-B-3-1	76.9	74.2	2449	77	100	6114	87	102	4875	89	107	7748
IR 91648-B-238-B-1-1	81.0	81.0	2370	81	100	9605	92	97	4365	90	118	9031
IR 91648-B-238-B-2-1	80.5	89.0	3153	83	104	6711	90	99	4999	92	119	10108
IR 91648-B-319-B-2-1	81.5	83.8	2303	83	104	8483	93	102	5179	91	114	7348
IR 91648-B-319-B-2-2	81.0	79.3	1690	84	95	8161	92	104	5431	91	119	10695
IR 91648-B-31-B-1-1	81.7	77.3	2111	80	96	6036	86	92	4095	84	107	7592
IR 91648-B-356-B-2-1	83.5	78.2	1955	83	104	10553	91	98	5073	91	118	8480
IR 91648-B-59-B-1-1	81.6	79.8	2138	81	100	8040	91	101	5041	91	123	9574
IR 91648-B-59-B-3-1	80.3	88.8	2669	83	95	7265	94	92	5143	91	119	9015
IR 91648-B-59-B-7-1	81.5	79.8	2291	82	98	7499	90	97	4996	91	118	8678
IR 91648-B-89-B-10-1	81.1	84.3	2165	85	96	8255	91	98	4267	90	114	9007
IR 91648-B-89-B-1-1	81.6	81.2	2211	83	98	10111	90	96	4150	91	123	10189
IR 91648-B-89-B-12-1	81.1	88.8	2006	83	104	9081	92	100	4665	106	120	9550
IR 91648-B-89-B-3-1	81.4	76.2	1731	84	100	7143	91	97	4505	91	116	8026
IR 91648-B-89-B-4-1	80.8	84.2	2059	83	106	7176	93	98	4542	93	115	9565
IR 91648-B-89-B-8-1	80.1	85.3	2322	82	103	6036	91	102	5475	91	113	9546
Swarna	105	56	0	95	91	6857						
