# Supplementary material for: QTLs for Tolerance of Drought and Breeding for Tolerance of Abiotic and Biotic Stress: An Integrated Approach
Source: PLoS One. 2014 Oct 14;9(10):e109574. doi: 10.1371/journal.pone.0109574 (PMC4196913; doi:10.1371/journal.pone.0109574)
Supplement: File S1 — Standard evaluation system used for large scale screening of rice lines to determine their tolerance reaction rice blast (A), field management of lowland experiments with details of crop cultivation, fertilizer management, and management of insect pests and weeds (B). (PDF) [file pone.0109574.s003.pdf]

File S1A: Standard evaluation system used for large scale screening of rice lines to determine their tolerance reaction rice blast

Seeds of this population are planted in nursery beds where it is inoculated by natural inoculum with 3-4 lineages *Magnaporthe oryzae* **(Figure S1A-1)**. The plants showing tolerance to the strains based on the standard evaluation scores **(Table S1A-1).** Lines with scores between 0-4 are advanced further to the field where they are grown in well watered conditions.

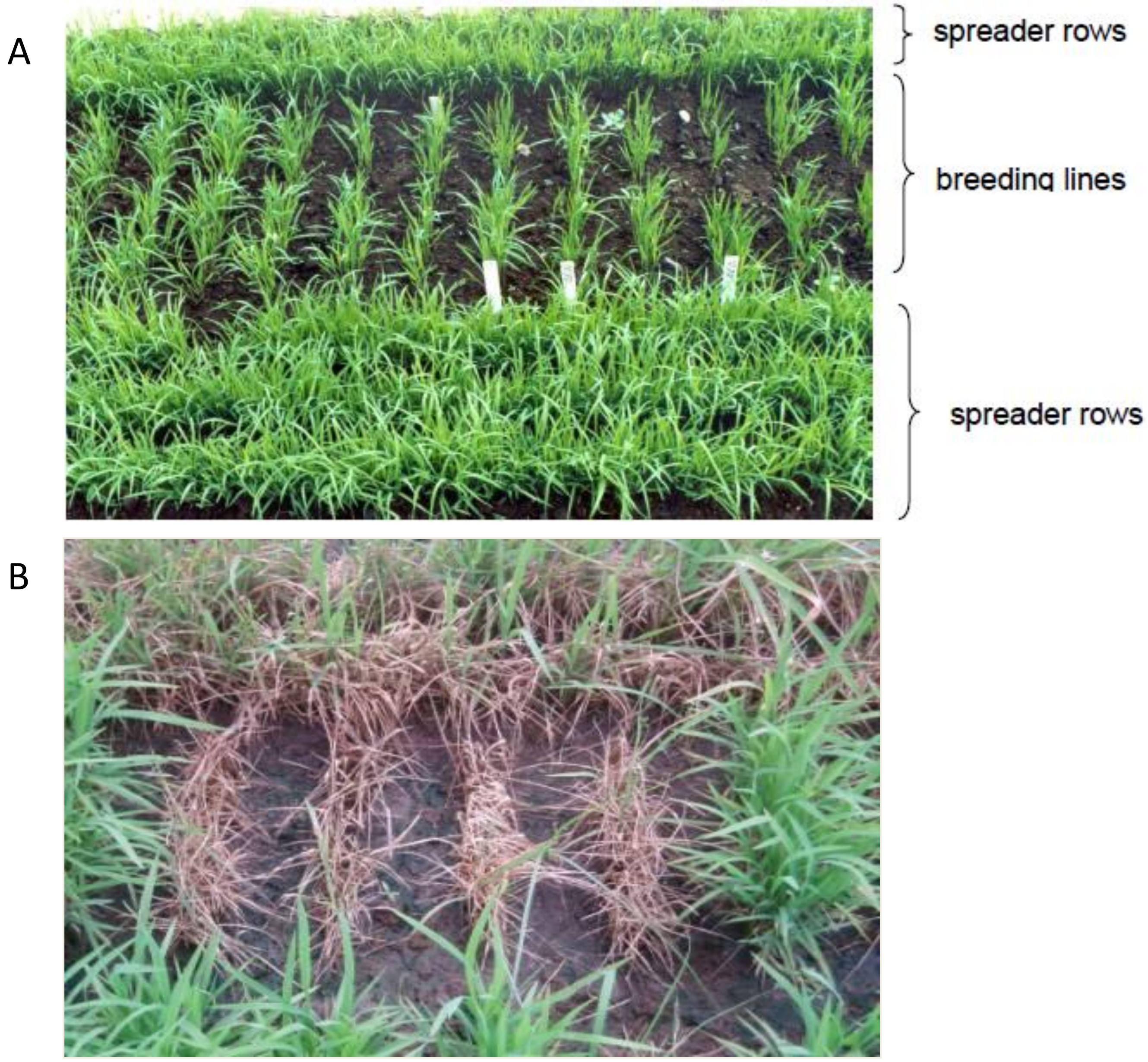

TableS1A-1: Standard evaluation system used to determine tolerance reaction of rice lines to rice blast

Scale	Description
0	No disease
1	Small brown specks of pin-point size
2	Small roundish to slightly elongated, necrotic grey spots, about 1-2 mm in diameter, with a distinct brown margin. Lesions are mostly found on the lower leaves
3	Lesion type is the same as in 2,but significant number of lesion are on the upper leaves
4	Typical susceptible blast lesions, 3 mm or longer, infecting less than 4% of the leaf area
5	Typical susceptible blast lesions, 3 mm or longer, infecting less than 4-10% of the leaf area
6	Typical susceptible blast lesions, 3 mm or longer, infecting less than 11-25% of the leaf area
7	Typical susceptible blast lesions, 3 mm or longer, infecting less than 26-50% of the leaf area
8	Typical susceptible blast lesions, 3 mm or longer, infecting less than 51-75% of the leaf area, many leaves dead
9	Typical susceptible blast lesions, 3 mm or longer, infecting more than 75% of the leaf area

File S1B: Field management of lowland experiments with details of crop cultivation, fertilizer management and management of insect pests and weeds

For all lowland trials, seeds were sown in a raised-bed nursery and 21-day-old seedlings were transplanted to the main field, with each hill containing one seedling. Basal application equivalent to 40 kg ha^{-1} phosphorus (P) and potash (K) was provided at the time of transplanting in the form of superphosphate and potassium chloride while 120 kg ha^{-1} nitrogen (N) was applied in the form of ammonium sulfate in three splits of 30, 45 and 45 kg ha^{-1} at 10, 20 and 45 days after transplanting, respectively, in the dry season while in wet-season trials the fertilizer doses decreased to 90, 25 and 20 kg ha^{-1} of N, P and K, respectively. Weeds were controlled by the application of post-emergence herbicide Sofit (pretilachlor ± safener, 0.3 kg a.i. ha^{-1}) at 4 days after transplanting while hand weeding was carried out in later stages. For insect control, Furadan (carbofuran, 1 kg a.i. ha^{-1}) was applied at 5 DAT, followed by Cymbush (cypermethrin, 1 l ha^{-1}) ± Dimotrin (cartap hydrochloride, 0.25 kg a.i. ha^{-1}) at 16 DAT. Bayluscide (niclosamide, 0.25 kg a.i. ha^{-1}), a molluscicide, was applied to control snails. Approximately 5 cm of standing water was maintained in stress trials until 30 days after transplanting. The fields were then drained for stress initiation and surface irrigation was provided only when the water table fell below 100 cm and 75% or more of the lines showed severe leaf rolling. Standing water was maintained up to 10 days before harvest for non-stress trials.
